# 3D feasibility of 2D RNA–RNA interaction paths by stepwise folding simulations

**DOI:** 10.1261/rna.079756.123

**Published:** 2024-02

**Authors:** Irene K. Beckmann, Maria Waldl, Sebastian Will, Ivo L. Hofacker

**Affiliations:** 1 Department of Theoretical Chemistry, Faculty of Chemistry, University of Vienna, 1090 Wien, Austria; 2 Vienna BioCenter PhD Program, Doctoral School of the University of Vienna and Medical University of Vienna, A-1030 Vienna, Austria; 3 Vienna Doctoral School in Chemistry (DoSChem), University of Vienna, 1090 Vienna, Austria; 4 Center for Anatomy and Cell Biology, Medical University of Vienna, 1090 Vienna, Austria; 5 LIX - Batiment Turing, Ecole Polytechnique, 91120 Palaiseau, France; 6 Faculty of Computer Science, Research Group Bioinformatics and Computational Biology, University of Vienna, 1090 Vienna, Austria

**Keywords:** RNA–RNA interaction, folding pathways, steric feasibility, coarse-grained folding simulation

## Abstract

The structure of an RNA, and even more so its interactions with other RNAs, provide valuable information about its function. Secondary structure-based tools for RNA–RNA interaction predictions provide a quick way to identify possible interaction targets and structures. However, these tools ignore the effect of steric hindrance on the tertiary (3D) structure level, and do not consider whether a suitable folding pathway exists to form the interaction. As a consequence, these tools often predict interactions that are unrealistically long and could be formed (in three dimensions) only by going through highly entangled intermediates. Here, we present a computational pipeline to assess whether a proposed secondary (2D) structure interaction is sterically feasible and reachable along a plausible folding pathway. To this end, we simulate the folding of a series of 3D structures along a given 2D folding path. To avoid the complexity of large-scale atomic resolution simulations, our pipeline uses coarse-grained 3D modeling and breaks up the folding path into small steps, each corresponding to the extension of the interaction by 1 or 2 bp. We apply our pipeline to analyze RNA–RNA interaction formation for three selected RNA–RNA complexes. We find that kissing hairpins, in contrast to interactions in the exterior loop, are difficult to extend and tend to get stuck at an interaction length of 6 bp. Our tool, including source code, documentation, and sample data, is available at www.github.com/irenekb/RRI-3D.

## INTRODUCTION

A large part of the transcripts in organisms are noncoding RNAs (ncRNAs). They perform diverse functions as regulatory elements for essential cellular processes in all domains of life (Shabalina and Koonin 2008; Waters and Storz 2009). These RNA functions crucially depend on their structure as well as their interaction with other RNAs. Experimental studies of kinetics and folding pathways for RNA–RNA interactions are challenging and time consuming. Therefore, computational tools that model the formation of RNA–RNA interactions offer an appealing alternative for studying these interactions.

In many cases, the level of secondary (2D) structure is sufficient to understand the function of an RNA. Computationally efficient tools for predicting reasonably accurate RNA 2D structures, such as are available in the ViennaRNA Package (Lorenz et al. 2011), are widely used. Further, there are extensions to predict RNA–RNA interactions like RNAup (Mückstein et al. 2006), RNAcofold (Bernhart et al. 2006), pairfold (Andronescu et al. 2005), NUPACK (Fornace et al. 2020), AccessFold (DiChiacchio et al. 2015), or IntaRNA (Mann et al. 2017). These prediction tools silently assume that any 2D structure can also be realized in three dimensions (3D). While uncritical for noncrossing single molecule structures, this assumption is no longer valid for RNA–RNA interactions. Consequently, 2D interaction prediction tools tend to “over-predict” interactions, disregarding that long interactions are often sterically infeasible. Even worse, they are often kinetically inaccessible, since the pathway toward the full interaction will be obstructed by steric effects. This lack of steric and kinetic considerations compromises the accuracy of conventional tools.

RNA 3D structure prediction remains a challenging and computationally demanding problem. However, available tools are well able to model smaller structural changes and to test whether 2D RNA–RNA interactions structure motifs are sterically feasible in 3D. We therefore designed a pipeline based on 3D prediction tools that breaks up the simulation into small steps to shorten the simulation time. In the stepwise extension setup, the required conformational change is small, making computations more efficient and less sensitive to the limitations of 3D modeling tools. The approach mimics the formation pathway of RNA–RNA interactions and therefore tests whether the final state as well as intermediaries are sterically feasible, thus assuring that the final state is kinetically reachable. If the extension process stops due to steric hindrance, we can identify how long interactions can be formed (which is often shorter than predicted by 2D tools). Applying the pipeline to several interaction examples leads to insights into the types of feasible interaction structures, which in turn can be incorporated as constraints in 2D structure prediction methods or used as a postprocessing filter. Ideally, this will facilitate efficient larger scale screens on a 2D level, without sacrificing steric feasibility. In addition, the developed pipeline with all its features allows for the investigation of specific interactions and different scenarios of interaction formation in depth.

Concretely, we selected three known RNA–RNA interaction systems, where at least some experimental data are available for further study: CopA–CopT, a well-studied antisense interaction (Kolb et al.2000a,b); the HIV-1 dimerization initiation site (DIS) (Ennifar and Dumas 2006), for which a 3D structure is available; and the DsrA–*rpoS* small RNA–mRNA interaction (Wu et al. 2017).

**FIGURE 1.**
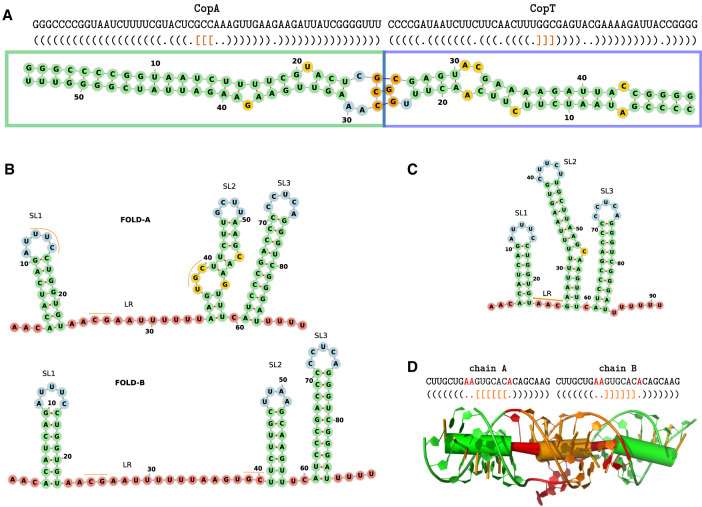
(*A*) Sequence and secondary structure representation of the 54 nt part of CopA (green box) and the corresponding 47 nt CopT (blue box) counterpart used in our simulation study. The three central residues of the hairpins (CCG/GGC) are the initial interaction site (marked in orange). (*B*) Fold-A and Fold-B of DsrA from Wu et al. (2017) in 2D structure representation. We label the stem–loops SL1–SL3 and the LR between SL1 and SL2. Fold-B opens the entire region from SL1 up to C40. (*C*) 2D DsrA consensus structure representation based on Rfam and predicted by the ViennaRNA package. The orange lines in (*B*) and (*C*) mark potential initial sites used later. (*D*) Sequence and dot-bracket representation of the HIV-1 DIS stem–loop interaction with the 6 nt kissing hairpin interaction marked in orange, and the conserved, unbounded, noncomplimentary nucleotides marked in red. The 3D representation below (visualized with PyMOL [Schrödinger 2022]) represents both: the individual nucleotides and the associated secondary structure in Ernwin-style, represented as cylinders. Thus, the green cylinders stand for the respective intramolecular stem in each chain, the orange one for the interaction, and the small red ones for the unbounded nucleotides. All 2D representations in this figure are generated with forna (Kerpedjiev et al. 2015a).

**CopA–CopT**. CopA is an antisense RNA that binds to its target CopT, which is part of the leader region of the *repA* mRNA, for plasmid replication control. The CopA–CopT interaction indirectly regulates the plasmid R1 replication by inhibition of the *RepA* translation. The CopA and CopT fragments used for our simulations and their initial binding at their complementary hairpins are shown in Figure 1A. On the CopT side, the hairpin contains a YUNR motif that induces a U-turn structure that promotes the initial binding (Franch et al. 1999). This system consists of two almost perfectly complementary chains (Supplemental Fig. S1). Although 2D prediction tools, without hesitation, predict the full-length duplex as the thermodynamically most stable state, it is not at all obvious (see Kolb et al. 2000b) that this state is very accessible starting from an initial (kissing hairpin) seed contact of the RNAs.
**HIV-1 DIS**. The HIV-1 dimerization initialization site is a strongly conserved feature in the 5′-untranslated region of the viral genome. Here, a slightly different experimental setup was used, because X-ray 3D structures are available. The 1ZCI (subtype F of the HIV-1 DIS) PDB structure shows a homodimer with each chain forming a 7 bp helix enclosing a 9 nt hairpin (Fig. 1D). The two hairpins interact to form a 6 bp intermolecular helix. Even though the monomers are not perfectly self-complementary, 2D interaction prediction tools will predict an extended interaction, interrupted by two 2 × 1 interior loops, spanning the full length of the 23 nt fragment. For our simulation, we chose the 1ZCI structure as the starting point and tested whether a refolding into the extended interaction is possible.
**DsrA–*rpoS*.** As a more complex example, we model the DsrA (downstream of rcsA)–*rpoS* interaction. DsrA is an Hfq-dependent small regulatory RNA in *Escherichia coli*. One of its targets is the *rpoS* mRNA, whose translation is up-regulated upon interaction with DsrA. Different structures have been proposed for DsrA, but all include stable stem–loop structures SL1 and SL3 at the 5′ and 3′ end. For the linker region (LR) and stem–loop SL2, we consider three different structures corresponding to Fold-A and Fold-B from Wu et al. (2017) (Fig. 1B), as well as the consensus structure from Rfam (Fig. 1C; Kalvari et al. 2020). The ViennaRNA package (Lorenz et al. 2011) predicts the Rfam structure as minimum free energy (MFE), followed by Fold-B as a near optimal alternative structure. Since Wu et al. (2017) suggest that DsrA needs to refold from Fold-A to Fold-B in order to interact with *rpoS*, we use this model to analyze how interaction formation depends on the starting structure and start site. To simplify the model and since little is known about the structure of *rpoS*, we model it as a 41 nt unstructured RNA.

### 3D modeling tools

Our approach is characterized by the use of coarse-grained 3D prediction and stepwise 3D simulation. To enable the 3D embedding of 2D interaction paths in reasonable computation time, we make use of two (comparatively fast) 3D modeling tools. One of them, Ernwin (Kerpedjiev et al. 2015b), is used to derive initial 3D structure sketches, while the other, SimRNA (Boniecki et al. 2015), is used to refine 3D structures and simulate folding paths. Crucial for the feasibility of our entire approach, both of these tools feature structure abstraction and coarse-graining (at different levels). Furthermore, we perform folding simulations only in iteration-limited steps to explore local structural neighborhoods. This keeps execution times low while improving control and reducing dependence on the exact details of SimRNA's coarse-grained simulation.

Ernwin generates 3D structures for a given secondary structure, applying a fragment assembly strategy on the level of loops and helices. It thus achieves very fast sampling, making it well suited to quickly sample candidate 3D structures in our approach.

SimRNA combines a knowledge-based potential with Monte-Carlo simulation to sample slightly coarse-grained structures. In contrast to Ernwin, it can predict completely novel structures, since it is not restricted to a fragment library. Both tools allow for translating their coarse-grained models back to atomic resolution. In the case of the Ernwin model, these back-translated models often contain local gaps and clashes that can be improved by a refinement step using SimRNA. SimRNA does not require a fixed secondary structure as input; however—essential for our stepwise approach—one can add (soft) constraints that steer the simulation toward a secondary structure. In particular, we use these soft constraints to specify the desired interaction base pairs. Soft constraints are implemented as an energy penalty in SimRNA, making all conformations that violate the constraint less likely. If a desired 2D structure is sterically impossible, SimRNA should not be able to fulfill the constraints. Thus, failure to fulfill the constraints is at least an indicator that a desired 2D structure cannot be embedded in 3D.

## RESULTS

### Pipeline

Our pipeline starts from a given path for RNA–RNA interaction formation in 2D. Such a path is a sequence of secondary structures, starting with an initial interaction, where each structure introduces a small step toward a target interaction (see, e.g., Supplemental Fig. S1). Then, it systematically attempts to find possible 3D explanations of the given path. Recall that not all 2D paths are expected to have (energetically and kinetically favorable) 3D support, such that the pipeline essentially tests 2D hypotheses. Operationally, the pipeline breaks up the computation into steps, each corresponding to one step of the 2D pathway. This avoids performing simulation of large structural changes which are computationally expensive and unreliable. We describe the general working mechanism along with various options that allow the pipeline to cover diverse concrete application scenarios.

To define the 2D path, one can either specify every 2D structure explicitly or let the path be generated systematically by extending an initial interaction toward its maximal bulge-free extensions or toward a given target structure; the latter possibly including bulges. The automatic extension can follow different schemes, alternatingly adding base pairs to the left and right or simultaneously adding a base pair to the left and the right of the interaction. To allow opening and refolding of the intramolecular structure, automatic path generation can moreover keep a spacer around the growing interaction site free from intramolecular base pair constraints. The structures along the 2D pathways are later used as soft constrains in the SimRNA simulations.

**FIGURE 2.**
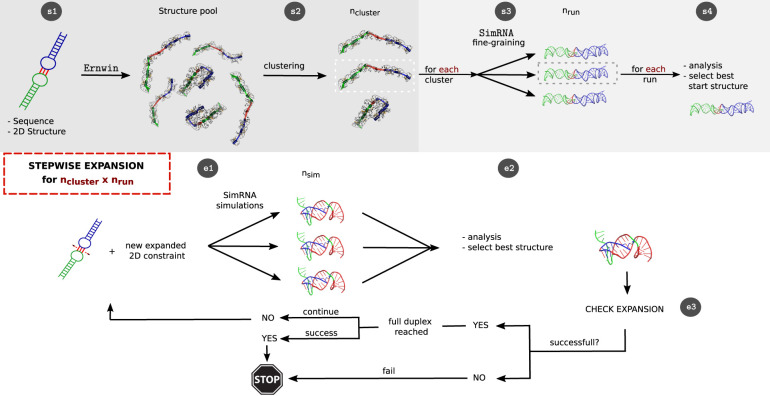
Graphical representation of the developed pipeline: An initial interaction is extended until the full duplex, a predefined target interaction, is reached or an extension of the interaction side is no longer possible due to steric and kinetic effects. (*s1*) Starting from a sequence and the corresponding secondary structure, 3D models are sampled using Ernwin and subsequently clustered (*s2*), yielding one representative per cluster. (*s3*) Each of these cluster representatives is relaxed in *n*_run_-independent SimRNA simulations. (*s4*) Each simulation is analyzed and a best structure is selected. Each of these *n*_cluster_ × *n*_run_ structures forms the start point for a stepwise expansion. In the first step, (*e1*) *n*_sim_ parallel, constrained SimRNA runs are performed. (*e2*) From the pooled structures the start structure for the next extension step is elected based on hierarchic 2D and 3D criteria. At the checkpoint (*e3*), we test whether the interaction region could indeed be expanded and terminate unsuccessful runs.

As shown in Figure 2, our pipeline is divided into the generation of a start structure (*s1*)–(*s4*) and the stepwise extension of the interaction site (*e1*)–(*e3*):

#### 1. Generation of a start structure

If a 3D start structure is provided (in PDB format), this step is omitted. Otherwise, 3D start structures are generated based on the sequence and an initial secondary structure.

(*s1*) A pool of coarse-grained start conformations is modeled using Ernwin.
(*s2*) The sampled conformations are clustered into *n*_cluster_ clusters based on their Ernwin fragments used. From each cluster, a representative is selected as the structure with the best energy.
(*s3*) For each of the *n*_cluster_ representatives, *n*_run_ short SimRNA simulations are performed to refine the structure, ensuring that gaps are closed and clashes are resolved. Constraints are used to ensure that the secondary structure is conserved during this refinement.
(*s4*) From each of the *n*_cluster_ × *n*_run_ SimRNA simulations, we select one structure that must (i) exactly match the specified initial 2D structure, and (ii) has the lowest SimRNA energy. These selected structures serve as start points for the stepwise extension.

#### 2. Stepwise extension

We now perform a series of stepwise extensions toward the full-length interaction, as described below. This procedure is performed independently for each of the start structures generated above.

(*e1*) *n*_sim_ short parallel SimRNA simulations (each of length *n*_step_) are performed with a secondary structure constraint designed to expand the interacting region. Structures from all *n*_sim_ simulations are pooled together.
(*e2*) From this pool of structures, the start structure for the next iteration is selected in a hierarchical fashion. To this end, we compare the secondary structure of the SimRNA model with the desired structure as defined by the constraints used in (*e1*) via the base pair distance. The selection is done by (i) comparing only the interaction region, (ii) considering the structure of the whole complex, and (iii) using the SimRNA energy to break ties.
(*e3*) If for a simulation the interaction does not expand despite the forcing constraint potential, we conclude that the maximum sterically possible interaction has been reached for this specific run and it stops. If the desired target interaction has been reached after an extension step, the simulation terminates with success. Otherwise, we continue with the next step of our 2D pathway at (*e1*).

The raw output of our pipeline is a large number of SimRNA trajectories that are available for further analysis. In order to facilitate the analysis of the large number of sampled 3D structures, each 3D structure is translated back into a 2D structure, i.e., a list of base pairs, including noncanonical base pairs recognized by the SimRNA_trafl2pdb tool. The main output of the RRI-3D pipeline are tab-separated files with statistics on these 2D structures. A more detailed description of pipeline output is available in the supplemental List 1 and the GitHub project page at www.github.com/irenekb/RRI-3D.

### Simulation results for model systems

#### CopA–CopT

**FIGURE 3.**
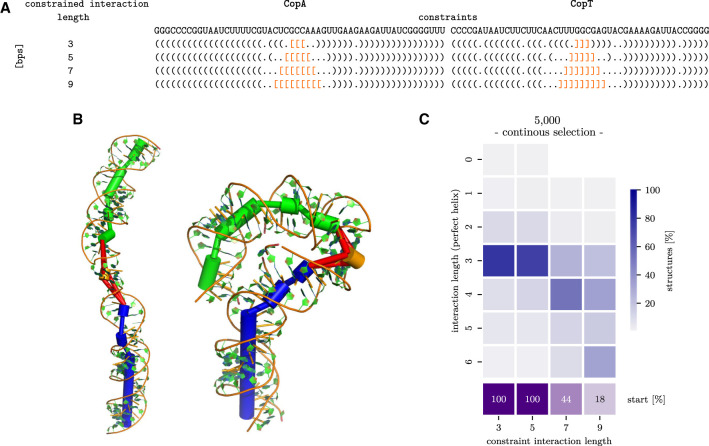
(*A*) CopA–CopT sequence and the dot-bracket representation (interaction in orange) of the constrained 2D path (full 2D path in Supplemental Fig. S1). (*B*) Two representative starting clusters with very different 3D conformations. CopA is shown in green, CopT in blue, and the interaction region in orange. (*C*) Summary of simulation results for *n*_step_ = 5000: Each column of the heatmap represents one extension step (defined by the length of the interaction constraint). Each column shows the histogram of observed interaction lengths (blue color gradient). In the first two extension steps, most structures retain the initial interaction length of 3 bp; 6 bp interactions become prevalent in the last extension step. The last row (purple) reports, for every extension step, the percentage of the (originally) 50 runs that successfully extended in all previous steps (i.e., passed checkpoint [*e3*]) and are still active. Thus, in the second extension step (constraint length 5), only 44% of runs manage to extend the interaction and proceed to the next extension step (7 bp). Only 18% of the runs reach the final extension step (9 bp), and none of these extends even further.

We used the start conformation proposed in Kolb et al. (2000b), consisting of the two stem structures with three defined interaction base pairs as interaction start. From the initial Ernwin simulations, we derived *n*_cluster_ = 10 clusters and started the extension simulation with *n*_run_ and *n*_sim_ = 5. The stepwise extension part of the pipeline (*e1–e3*) was repeated three times with different *n*_step_ (5000, 10,000, 100,000). In the course of the stepwise extension, both intra- and intermolecular pairs from the corresponding structure in the 2D path (Fig. 3A; Supplemental Fig. S1) were used as SimRNA soft constraints as well as in the selection process. Adding intramolecular constraints is helpful to avoid helix ends opening during the simulation and focuses on exploring the dynamics of the interaction region. Additionally, two different stop criteria (*e3*) were compared. In the first setting (I), the pipeline stops as soon as the simulation was not able to further extend the interaction as a perfect helix. In the second setting (II), the pipeline continues if the interaction region can be extended, even at the expense of interruptions such as bulges or small interior loops. The latter procedure allows flexibility within the interacting region and thus can proceed to longer interactions. Detailed descriptions of the simulation parameters, including the settings for the examples presented here, are available on the RRI-3D website. All starting structures contained an initial interaction of 3 bp; however, the structures of the 10 clusters generated by Ernwin differed considerably (see, e.g., Fig. 3B). Especially for short SimRNA simulations (*n*_step_ = 5000, 10,000), the starting cluster strongly affected the ability to extend the interaction site. While for some start clusters a 6 bp interaction formed spontaneously within the very first SimRNA simulation, others did not reach the 6 bp stage at all unless *n*_step_ was increased. Results for shortest (*n*_step_ = 5000) SimRNA simulations are shown in Figure 3C and Supplemental Figure S2A.

**FIGURE 4.**
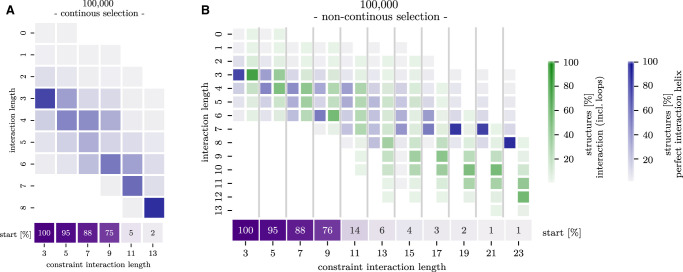
Distribution of CopA–CopT interaction lengths for two different pipeline settings and long SimRNA runs (*n*_step_ = 100,000); see Figure 3 for description. (*A*) Setting I, extensions form perfect helices. (*B*) Setting II, with loops in the interaction region allowed. Histograms are shown for perfect interaction helices (blue), as well as interactions with loops (green).

Pipeline runs with *n*_step_ = 5000 or 10,000 SimRNA steps hardly differ (Supplemental Fig. S2B). Long SimRNA simulations with *n*_step_ = 100,000 allow nearly every cluster to extend to 6 bp continuous interaction (Fig. 4A). In setting I, some runs even reach 7–8 bp interaction length; however, this only happens when the SimRNA constraint has already been extended to 11–13 intermolecular base pairs. Indeed, all structures with more than 6 bp interaction exhibit very high constraint penalties; see Supplemental Figure S2C. This suggests that an interaction length of 6 bp presents an optimum that can only be overcome by very strong constraints and indicates that quite some effort is needed for further folding. Setting II, i.e., with loops and bulges within the interaction region, allows the formation of longer interactions. While the interacting region could contain up to 13 bp (with a peak at 12 bp), no uninterrupted helices longer than 8 bp were observed (Fig. 4B). The results of our simulations are consistent with the CopA–CopT pathway proposed by Kolb et al. (2000b), in which an interior loop is formed in the interaction site in order to allow longer interaction. Even in this setting, most runs terminate at an interaction length of 6 bp. Again, longer interaction regions require more base pair constraints (13–23 bp) and thus stronger constraints (Supplemental Fig. S3). Moreover, the relative orientation of the CopA and CopT stems can influence how easily interactions can be extended; see Supplemental Figure S4. An analysis of the SimRNA energies (Supplemental Fig. S3) shows that 6 bp conformations are energetically favorable with small constraint violation energies, while longer interactions exhibit higher constraint energies. Moreover, conformations with interacting regions longer than 6 bp have consistently worse energies, indicating that these structures could be the result of strong constraints, rather than naturally forming conformations.

#### HIV-1 DIS

**FIGURE 5.**
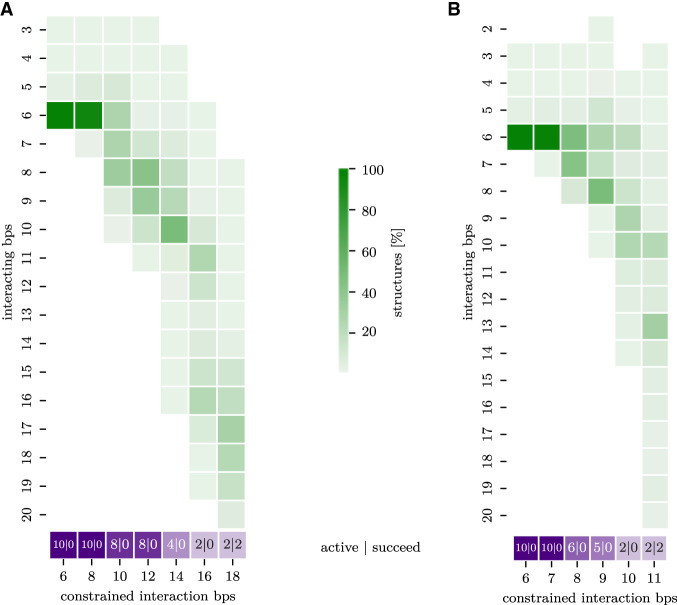
(*A*) Setting I. Symmetric interaction extension of the HIV-1 DIS interaction site with *n*_step_ = 1 million. Each column shows the histogram (green) of interaction lengths including loops (*y*-axis) at the corresponding constrained interaction length in bp (*x*-axis). For every elongation step (corresponding to a number of constrained interaction base pairs), the purple box reports firstly the number of still active runs (from a total of 10); and secondly, the number of runs that successfully reach the full duplex. (*B*) Setting II with an asymmetric interaction extension.

Since the full extended interaction target of the HIV-1 DIS segment includes two loops, we specify the start and end conformation for our pipeline. The enclosing intramolecular helices were left unconstrained (see Supplemental Fig. S5 for the full 2D path). Instead of using Ernwin, we started from the 1ZCI PDB structure (and thus have *n*_cluster_ = 1), but increased *n*_run_ and *n*_sim_ to 10 in order to increase 3D structure diversity. With the previously used settings for SimRNA simulations (*n*_step_ = 10,000 or 100,000), we were unable to extend the initial 6 bp kissing interaction. We conjecture three causes: the interaction length of 6 bp appears to be especially stable for kissing hairpins; the interior loop needs to be bridged in order to reach longer interactions (Fig. 1D); and the PDB structure provides a particularly good initial 3D fold. We therefore performed additional pipeline runs with extremely long SimRNA simulations of *n*_step_ = 1 million, and two different expansion modes were compared. In setting I, each extension step elongates the interaction region by 1 bp in both directions (symmetric); see Supplemental Figure S5A for the given constraints and Figure 5A for the results. In setting II, we extend only in one direction until the maximum extent is reached (asymmetric); see Supplemental Figure S5B and Figure 5B. With these extremely long SimRNA simulations, two out of 10 runs were able to form an extended duplex for both settings. In both settings, two intermediates with an interaction length of 8 bp (especially in setting I) and around 12 bp can be observed. After the second intermediate state, the enclosing helices unfold completely and the full-length interaction is formed spontaneously. A notable difference between the two settings is that for setting I, the simulation passes through intermediates with very high constraint energies (i.e., many unsatisfied base-pairing constraints); see Supplemental Figure S5.

At interaction length 12, the enclosing stem has shrunk to 3–4 bp in length, and further extension leads to the complete unfolding of the enclosing stem. This unfolding of the remaining enclosing stem corresponds to the final energy barrier, after which the extended duplex can be reached. Looking at the SimRNA energies (Supplemental Fig. S5) and stem lengths, we observe that the second intermediate state has its energy minimum at an interaction length of 12 and a remaining enclosing stem of 3–4 bp. This seems to be the minimum length at which the enclosing stem remains stable. With the next extension, it unfolds completely, allowing it to reach the full extended duplex by a downhill walk on the energy landscape.

Note, however, that even with these settings eight out of 10 runs remain trapped close to the 6 bp state. Moreover, the PDB structure contains only a 23 nt long fragment of the HIV genome and therefore a shortened enclosing helix, which would be even more difficult to unfold in the context of the full genome.

#### DsrA–rpoS

**FIGURE 6.**
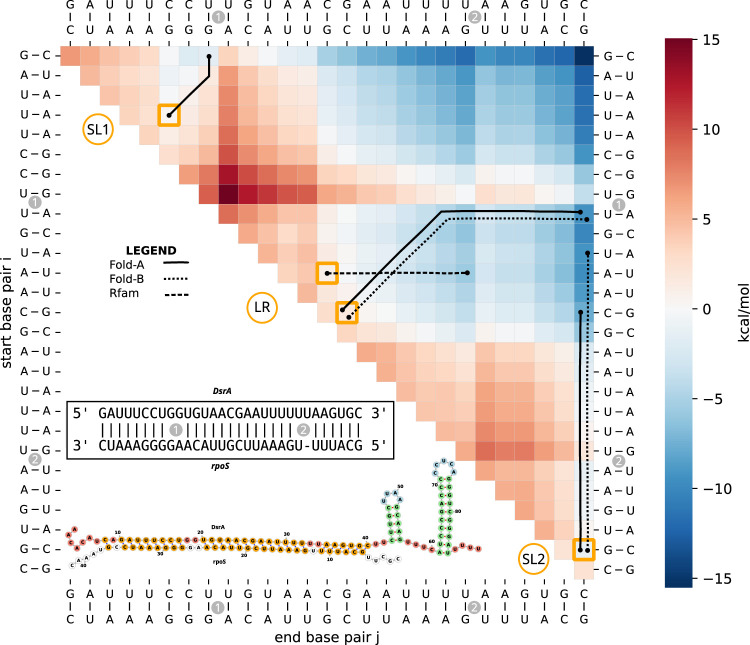
Interaction of DsrA–*rpoS* in *E. coli* as a direct path energy landscape following RRIkinDP (Waldl et al. 2023) and comparison of different formation scenarios. RRIkinDP, a novel 2D structure-based tool for studying RNA–RNA interaction formation on direct paths, was used to generate the energy landscape of structures along folding pathways from a first interaction base pair to the full DsrA–*rpoS* interaction. To study interaction formation, RRIkinDP considers intermediate interactions formed by consecutive interaction base pairs. Each intermediate interaction is therefore defined by the first and last interaction pair. For visualization, intermediate states can therefore be arranged in an upper triangular matrix, indexed by two enclosing pairs on the *x*- and *y*-axis. Each cell of the matrix represents an intermediate interaction, with minimal interactions consisting of a single base pair on the diagonal and the full, maximal length, interaction in the *upper right* corner. Cell colors depict the free energy Δ*G* on a red-to-blue scale. For details, see Waldl et al. (2023). The predicted 2D interaction for DsrA–*rpoS* appears in the *lower left* triangle, presented in two ways: the interaction site *above*, with interior loops marked as (1) and (2), and a 2D representation of the complete interaction complex *below*. Using the energy landscape, we identified three energetically favorable start sites (highlighted in orange): within the loop of the first stem–loop (SL1), within the LR, and within the bulge of the second stem–loop (SL2), as shown in Figure 1. From these sites, we generated direct folding paths in 2D, which were subsequently embedded in 3D using the RRI-3D pipeline. The resulting 3D pathways were projected back into 2D and are depicted as black lines within the heatmap. In the path, moving diagonally corresponds to extending the interaction on both sides, while moving up or to the right extends the interaction by 1 bp on the left or right, respectively. Different linestyles represent different initial folds of the DsrA SL2 structure (see legend and Fig. 1).

We selected the interaction between DsrA and *rpoS* as an example with different interaction formation scenarios that can be investigated with our pipeline. IntaRNA, a thermodynamics-based 2D interaction prediction tool, predicted an extended interaction, depicted in the lower half of Figure 6 (see also Wu et al. 2017). Specifically, we compared three different possible intramolecular start structures of DsrA (Fig. 1) as well as the influence of the initial contact point. Based on RRIkinDP (Waldl et al. 2023), the energy landscape (see heatmap in Fig. 6) of all possible intermediate interactions was computed in 2D. This provided three favorable start interaction sites with high accessibility which we selected for modeling using our 3D pipeline: (i) in the SL1 loop (Fold-A only), (ii) at the start of LR (all three conformations), and (iii) in the bulge of SL2 in Fold-A (respectively the end of LR in Fold-B). As for CopA–CopT, we ran our pipeline with *n*_cluster_ = 10, *n*_run_ = 5, and three different simulation lengths *n*_step_ = 5000, 10,000 and 100,000. In total, we tested six combinations of initial contact and starting structure; see Figure 1B,C. The corresponding 2D pathways used for SimRNA constraints are shown in Supplemental Figures S7–S10. Two-dimensional projections of representative folding paths for each scenario are also marked in the energy landscape (Fig. 6).

Out of the three start points, the hairpin of SL1 is clearly the worst. The 3 bp initial interaction spontaneously extends to 6 bp in length. However, all simulations terminated without fully unfolding the SL1 stem. Even with an increasing number of constraints, almost no interaction length >8 is sampled; see Supplemental Figure S11. This was somewhat expected from the energy barrier seen in the 2D landscape.

Simulations starting in LR quickly extend in the 3′ direction over the whole LR, regardless of whether Fold-A (Supplemental Fig. S13) or Fold-B (Supplemental Fig. S14) is assumed. When starting in Fold-A the interaction unwinds the lower part of SL2, suggesting that a refolding into Fold-B would eventually happen. Even though the constraints are extended symmetrically in both directions, the observed interaction region grows much more quickly in the 3′ direction, while <10% of runs manage to extend into SL1. For the Rfam structure, our initial contact is wedged in between SL1 and SL2. Since SL2 is much weaker, we used a 2D path that extends toward SL2 (Supplemental Fig. S10). The simulations manage to partially unfold SL2 (Supplemental Fig. S15). Even though this version of SL2 consists of weak AU and GU pairs, it slows down interaction formation due to its length. Nineteen out of initially 50 simulations manage to at least dissolve the 6 bp helix until the bulge.

Starting with SL2 as the start site, Fold-B (Supplemental Fig. S12B) has a slight advantage compared to Fold-A (Supplemental Fig. S12A), since it avoids opening the additional 3 bp of SL2. However, the fraction of simulations that extend all the way to SL1 is almost the same. Only a single simulation manages to extend slightly into SL1.

In comparison, our simulations suggest that optimal interaction formation should start within the LR of Fold-A or Fold-B. In contrast to Wu et al. (2017), we find that starting in Fold-A works almost equally well as starting in Fold-B and may even trigger refolding into Fold-B. The Rfam structure offers fewer highly accessible start points in LR and additionally, interaction formation is slower. In all cases, SL1 was too stable to be dissolved during our simulations. However, once a stable interaction has been formed within the LR region, the DsrA–*rpoS* complex will not dissolve and could eventually extend beyond SL1 on a timescale beyond what is covered in our simulations.

## DISCUSSION

Our presented approach enables detailed studies of potential 2D folding pathways for the formation of RNA–RNA interactions in 3D. To overcome the typically extreme computational cost of 3D structure prediction, we use coarse-grained 3D simulations and, moreover, construct interactions step-by-step in a series of length-limited simulation runs. Following a predefined 2D path of secondary structures, each step corresponds to an elementary extension of the interaction by 1 or 2 bp. This allows us to simulate large structural changes that are inaccessible for atom-level simulations such as molecular dynamics. Moreover, it allows us to perform a sufficiently large number (*n*_cluster_ × *n*_run_ × *n*_sim_) of independent simulations in order to sample diverse starting conformations and alternative pathways. As an example, a typical CopA–CopT simulation with *n*_cluster_ = 10, *n*_run_ = 5, *n*_sim_ = 5, *n*_step_ = 10,000 took about 19 h, using up to 10 cores.

In contrast to models that only consider secondary structure, this allows us to identify likely obstacles in the folding path due to steric hindrance. A paradigmatical example is the interaction (“kissing”) between two hairpins as in the case of CopA–CopT. In this case, due to the perfect complementarity, secondary structure tools would typically extend any initial contact up to the full duplex, completely neglecting steric effects. Radically changing the picture, our pipeline reveals that extending the interaction beyond the 6 bp stage becomes increasingly difficult; or even impossible while maintaining perfect stacking: rarely, we observed kissing hairpin interactions of more than 8 bp; all of them contained bulges or small interior loops, presumably in order to relieve strain. This is consistent with the structures proposed by Kolb et al. (2000b) on the basis of mutation and probing experiments. We find similar behavior for the HIV-DIS interaction, which also forms a kissing hairpin. In this case, a few simulations reach a full duplex, but this is only possible because we simulate a short fragment of the HIV genome, and thus the enclosing helix is short enough to completely unfold. A more complex example is represented by the DsrA–*rpoS* interaction, where we show that our approach can be used to compare different scenarios of interaction formation, such as starting from different monomer structures or different initial contact points. Our observation that kissing hairpins are difficult to extend into long interactions is consistent with the fact that there are almost no known natural kissing hairpin structures (including those in pseudoknots) with more than six intermolecular pairs. Using a modified Forgi script (Thiel et al. 2019) and the nonredundant 3D structure set of Leontis and Zirbel (2012), we found that among all kissing hairpin pseudoknots with genus 1 (as defined by Reidys et al. 2011), out of 44 pseudoknots only the group II intron (PDB 3IGI by Toor et al. 2009) with an interaction length of 7 bp is longer, but this occurs between a hairpin loop and an interior loop. We note that the pipeline can also be used to test the 3D feasibility of pseudoknots predicted on the basis of secondary structure, as done by Gosavi et al. (2022).

An attractive future application of our pipeline would be to postprocess and evaluate conventional secondary structure-based interaction predictions. Here, an RNA targeting screen as performed by fast 2D tools like RISearch2 (Alkan et al. 2017) could be scrutinized for 3D steric feasibility by our pipeline. Adding to this idea, insights from example systems can be used to flag 2D-based predictions that call for further investigation. In particular, predictions relying on long (>6 bp) intermolecular helices within stable intramolecular structures should be contested. Conversely, long interactions are uncritical, even favorable in exterior loops.

Finally, our findings motivate the future improvement of 2D-based tools by incorporating 3D features (e.g., context-dependent length restrictions) or even tighter integration with 3D modeling.

## DATA DEPOSITION

Python source code for our pipeline, including documentation as well as data and scripts to analyze the three example systems, is available at https://github.com/irenekb/RRI-3D.

## SUPPLEMENTAL MATERIAL

Supplemental material is available for this article.

## Figures and Tables

**Irene K. Beckmann:**
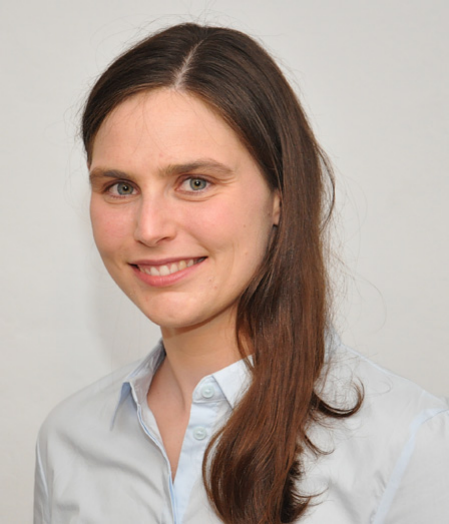


## MEET THE FIRST AUTHOR

***Meet the First Author(s)* is an editorial feature within *RNA*, in which the first author(s) of research-based papers in each issue have the opportunity to introduce themselves and their work to readers of *RNA* and the RNA research community. Irene Beckmann is the first author of this paper, “3D feasibility of 2D RNA–RNA interaction paths by stepwise folding simulations.” Irene is a PhD candidate in Ivo L. Hofacker's group at the University of Vienna, which specializes in bioinformatics. Irene's focus is on the development of computational tools for the prediction and analysis of RNA interactions and their dynamics in a three-dimensional context, and she recently explored the 3D feasibility of 2D RNA–RNA interaction paths through stepwise folding simulations.**

**What are the major results described in your paper and how do they impact this branch of the field?**

The main outcome of this research is the development of the computational pipeline “RRI-3D.” The pipeline evaluates whether a proposed RNA structure interaction pathway is sterically feasible and reachable in 3D. This will contribute to a more accurate prediction of RNA–RNA interactions.

**What led you to study RNA or this aspect of RNA science?**

This research provides a better understanding of the functionality and regulatory mechanisms of RNA interactions and improves the prediction of regulatory targets. Our research on RNA interactions was motivated by the need to improve the accuracy of existing secondary structure-based tools for predicting RNA–RNA interactions by considering the 3D structure of RNAs.

**During the course of these experiments, were there any surprising results or particular difficulties that altered your thinking and subsequent focus?**

When we ran our pipeline with a higher number of clusters—in other words, by expanding the 3D space covered—we found that the results were much more diverse than initially expected. The analysis and classification of the large and complex amount of 3D RNA interaction data taught me the importance of breaking down complex tasks into manageable parts and being open to new approaches.

**What are some of the landmark moments that provoked your interest in science or your development as a scientist?**

While completing my MSc in molecular biology, I became interested in RNA research. Realizing the impact of RNA on various aspects of life, and the many open questions that remain regarding its nature, inspired me to begin studying RNA to gain a better understanding.

**If you were able to give one piece of advice to your younger self, what would that be?**

I would emphasize the importance of accepting setbacks and seeing them as a valuable learning experience, as they are also an essential part of the complex puzzle that we are trying to piece together with our research.

**Are there specific individuals or groups who have influenced your philosophy or approach to science?**

The teams in the Bujnicki lab at the International Institute of Molecular and Cell Biology in Warsaw and in the Hofacker lab at the Institute of Theoretical Chemistry in Vienna have mainly influenced my approach to science. I learned how important it is to have good and user-friendly documentation of the programs, ensuring accessibility to people outside the field of computational science. In addition, working with the other authors has taught me the importance of incorporating different perspectives, allowing me to explore problems from multiple perspectives and encourage personal growth.
